# Olfactory detection of a bacterial short-chain fatty acid acts as an orexigenic signal in *Drosophila melanogaster* larvae

**DOI:** 10.1038/s41598-017-14589-1

**Published:** 2017-10-27

**Authors:** Ana Depetris-Chauvin, Diego Galagovsky, Charlene Chevalier, Gerard Maniere, Yael Grosjean

**Affiliations:** 1 Centre des Sciences du Goût et de l’Alimentation, AgroSup Dijon, CNRS, INRA, Univ. Bourgogne Franche-Comté, F-21000 Dijon, France; 2 Present Address: Department of Evolutionary Neuroethology, Max Planck Institute for Chemical Ecology, 07745 Jena, Germany

## Abstract

Microorganisms inhabiting fermenting fruit produce chemicals that elicit strong behavioral responses in flies. Depending on their ecological niche, individuals confer a positive or a negative valence to a chemical and, accordingly, they trigger either attractive or repulsive behaviors. We studied the case of bacterial short-chain fatty acids (SCFA) that trigger opposite behaviors in adult and larvae of *Drosophila melanogaster*. We determined that SCFA-attractive responses depend on two larval exclusive chemoreceptors, Or30a and Or94b. Of those SCFA, propionic acid improves larval survival in suboptimal rearing conditions and supports growth. Olfactory detection of propionic acid specifically is sufficient to trigger feeding behaviors, and this effect requires the correct activity of Or30a^+^ and Or94b^+^ olfactory sensory neurons. Additionally, we studied the case of the invasive pest *Drosophila suzukii* that lives on undamaged ripe fruit with less SCFA production. Contrary to *D. melanogaster*, *D. suzukii* larvae show reduced attraction towards propionic acid, which does not trigger feeding behavior in this invasive species. Our results demonstrate the relevance of propionic acid as an orexigenic signal in *D. melanogaster* larvae. Moreover, this study underlines that the changes on ecological niche are accompanied with alterations of olfactory preferences and vital olfactory driven behaviors.

## Introduction

Chemoreception, the detection of environmental chemicals without the need of internalization and metabolization, is the most ancient sense and it is present in all living organisms. Accurate identification of chemicals followed by an internal assessment of sensory stimuli’s hedonic value, serves to explore the environment in search of beneficial compounds and helps avoid toxic components^1^. The innate hedonic value of chemicals is highly dependent on the ecological niche occupied by each species, with some chemicals being attractive for some species but indifferent or even repulsive for others^2,3^.

In terrestrial metazoans, chemoreception evolved to two anatomically and functionally distinguishable systems: olfaction and taste. Although both chemoreceptive modalities are essential for animal survival, the sense of smell plays a pivotal role in most terrestrial animals since it allows the detection of an immense variety of small volatile molecules (i.e. odorants) from a long distance. The insect *Drosophila melanogaster* represents an excellent model organisms to study odor-evoked behaviors and olfactory coding in a much simpler sensory system than that of mammals. In the adult fruit fly, primary detection is performed by heterodimeric chemoreceptors expressed in olfactory sensory neurons (OSNs) at the antenna and maxillary palps. These chemoreceptors belong to the gene family of olfactory receptors (OR), ionotropic receptors (IR) and to a lesser extent, gustatory receptors (GR). OR dimers consist of one common subunit (the coreceptor Orco) and a second subunit that provides the ligand-specificity^4,5^. For the case of IRs, specific IR subunits are coupled with one of 3 coreceptors: Ir8a, Ir25a, or Ir76b^6,7^. The larval system is even simpler, with only 21 OSNs located in one olfactory structure, the dorsal organ (DO), and with only members of the OR family accurately established as olfactory receptors^8,9^.

In addition to the benefits of a simpler olfactory system, the amazing repertoire of genetic tools together with the extensive information available on fly ecology makes *Drosophila melanogaster* an excellent organism to study odor perception in an ecological context^10^. *Drosophila melanogaster* feeds, mates, and lays eggs on decomposing fruit^11^. Fermenting fruit not only represents a softer medium for females to oviposit and for larvae to nourish, but also provides a variety of microorganisms that support larval growth and survival^12,13^. Consistent with these essential roles of microorganisms, adult and larvae of *Drosophila melanogaster* display attraction to several yeast and bacteria derived-odors^10^. Among the metabolites produced by yeast and bacteria during fruit decomposition, short-chain fatty acids (SCFA) are of particular interest. Several studies have demonstrated that SCFA produced from dietary fiber fermentation, *i.e*. acetic, propionic, and butyric acid, exert multiple beneficial effects on mammalian energy metabolism^14^. In the case of the fly environment, many SCFA are commonly present in fruits, with acetic and, to a lesser extent, butyric acid being the most abundant^15–18^. Interestingly, the levels of bacterial SCFA, *i.e*. propionic and butyric acid, significantly increase after secondary fermentation on rotting fruit, the natural habitat of *Drosophila melanogaster* ^19,20^. Behavioral studies suggest that acetic, propionic, and butyric acid are complex olfactory signals since they can trigger aversive behaviors in adults^21^, while they lead to attraction in larvae^22,23^. The olfactory aversive responses in adults are mediated mainly by detection through the Ir8a-coupled receptors, Ir75a, Ir75abc, and Ir64a^6,7,21^, and to a lesser extent ORs and Ir76b^24,25^. On the other hand, SCFA-attractive responses in larvae have only been briefly described. There is no conclusive information of the chemoreceptors relevant for behavioral responses nor an analysis of the physiological consequences of SCFA perception at the larval stage. The behavioral shift observed between SCFA-responses in larvae and adults might be a response to a specific beneficial role of these compounds at the larval stage. In addition, most works focus on acetic acid perception in adults^26,27^, while the bacterial SCFA, propionic and butyric acid, and its effects over behavior remains mostly unexplored.

In this paper we studied odor-evoked behaviors in response to bacterial SCFA at the larval stage. We first established that the larval receptor Or30a is essential for olfactory responses of the two bacterial SCFA, while the larval receptor Or94b is implicated only in propionic attraction. Furthermore, we demonstrated that propionic acid improves larval survival in suboptimal rearing conditions and supports growth by promoting feeding behavior. Finally, we explored behavioral responses toward propionic acid in a related species that exploits a different ecological niche with lower exposure to bacterial SCFA, the recent invasive pest *Drosophila suzukii* ^28^. We determined that propionic acid is less attractive and is not an orexigenic signal for larvae of *D. suzukii*, suggesting that in this species the ecological shift led to a loss of the propionic acid beneficial effects.

## Results

### Bacterial SCFA are attractive to foraging larvae and they are detected by specific ORs

**Figure 1:**
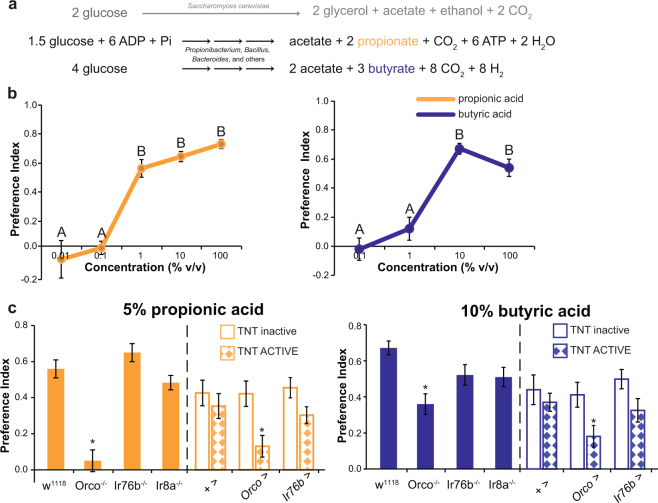
Foraging larvae are attracted to bacterial SCFA, and the attraction depends on olfactory receptors. (**a**) Biochemical process of glucose fermentation that produces acetate, propionate, and butyrate. Of those, propionate and butyrate are exclusively produced by bacteria. (**b**) Dose-response curves for propionic (LEFT) and butyric acid (RIGHT) in control (*w* ^1118^) 3^rd^ instar foraging larvae. Different letters indicate statistically significant differences with a p < 0.05 (non-parametric test Kruskal-Wallis). (**c**) Preference index at 5% propionic (LEFT) and 10% butyric acid (RIGHT). Mutation of the general olfactory coreceptor Orco or inactivation of all OSNs significantly reduced the attraction towards propionic and butyric acid. *Orco* null mutants displayed complete indifference towards 5% propionic acid while a residual attraction was still evident with 10% butyric acid (t-Test with null Hypothesis PI = 0, p = 0.4557 and p < 0.0001 for propionic and butyric acid, respectively). Mutants for *Ir76b* and *Ir8a* behaved as controls. For the null mutants *Orco* ^−/−^, *Ir76b* ^−/−^, and *Ir8a* ^−/−^ the “*” indicates statistically significant differences with the control group *w* ^1118^ (One-way ANOVA with a Tukey post-hoc test, p < 0.05). In the case of TNT neuronal inactivation, the “*” indicates statistically significant differences with respect of the two genetic controls, UAS-TNT ACTIVE/ + and the specific driver line crossed with TNT inactive (Two-way ANOVA with a Duncan post-hoc test, p < 0.05). From left to right the complete genotypes are: *w* ^1118^, *Orco* ^−/−^ = *Orco* ^2^, *Ir76b* ^−/−^ = *Ir76b* ^2^, *Ir8a* ^−/−^ = *Ir8a* ^1^ mutants, +>TNT inactive, +>TNT ACTIVE, *Orco* > TNT inactive, *Orco* > TNT ACTIVE, *Ir76b* > TNT inactive, and *Ir76b* > TNT ACTIVE. Data represents the average ± standard error of the mean and an average of 20 independent replicates were considered for each experimental group.

In the context of the fly environment, SCFA are produced after sugar fermentation by microorganisms inhabiting rotting fruit. Of the most biologically relevant SCFA, acetic acid is produced by yeast, *e.g. Saccharomyces cerevisae*, and bacteria (*Acetobacter* and *Gluconobacter*), while propionic and butyric acid are exclusively produced by bacteria like *Propionibacterium*, *Bacillus*, and *Bacteroides* via a complex series of reactions^29^ (Fig. 1a). To characterize odor-evoked behavioral responses towards bacterial SCFA in larvae we performed dose-response curves for propionic and butyric acid using a classical two choice chemotaxis assay^23^. Control third instar foraging larvae (*w* ^1118^) were highly attracted to the vapors of both propionic and butyric acid. In the case of propionic acid, control larvae responded already with the maximal preference index at 1% concentration; on the contrary, 1% butyric acid was not sufficient to trigger larval attraction and the maximal response was only observed once the concentration was increased to 10% butyric acid (Fig. 1b).

Next, we sought to establish the olfactory receptors responsible for the bacterial SCFA-attractive responses in larvae. Firstly, we analyzed propionic and butyric acid olfactory responses in mutant larvae for Orco, Ir8a, and Ir76b, the 3 olfactory coreceptors known to be relevant for propionic and butyric acid olfactory detection in adults^7,24,25^. For this, we analyzed null mutants for each coreceptor and we blocked chemical synapses in each OSNs type by targeted expression of tetanus neurotoxin light chain using the TNT line. *Orco* null mutants or neuronal inactivation of Orco^+^ neurons with TNT significantly reduced attractive responses towards propionic and butyric acid in larvae (Fig. 1c and Supplementary Fig. S1). Surprisingly, while *Orco* null mutants displayed complete indifference towards 5% propionic acid, at 1% propionic acid a residual attraction is still evident, suggesting that at lower concentration other family of olfactory receptors or even taste perception could be involved. Genetic control lines exhibited reduced attraction towards 1% propionic acid probably as an ectopic noxious effect of the TNT ACTIVE strain (Supplementary Fig. S1), so we continued the analysis of behavioral responses using 5% propionic acid. *Ir8a* null mutant larvae showed normal attractive responses towards both propionic and butyric acid, consistent with a lack of expression of this coreceptor at the larval stage^6^ (Fig. 1c). The coreceptor Ir76b is expressed both in larvae and adults in olfactory and taste organs^30–32^, and at the adult stage it is marginally involved in the detection of propionic and butyric acid at the antenna^25^. In larvae we found that *Ir76b* null mutants or neuronal inactivation of Ir76b^+^ neurons did not affect propionic nor butyric acid attractive responses (Fig. 1c).

**Figure 2:**
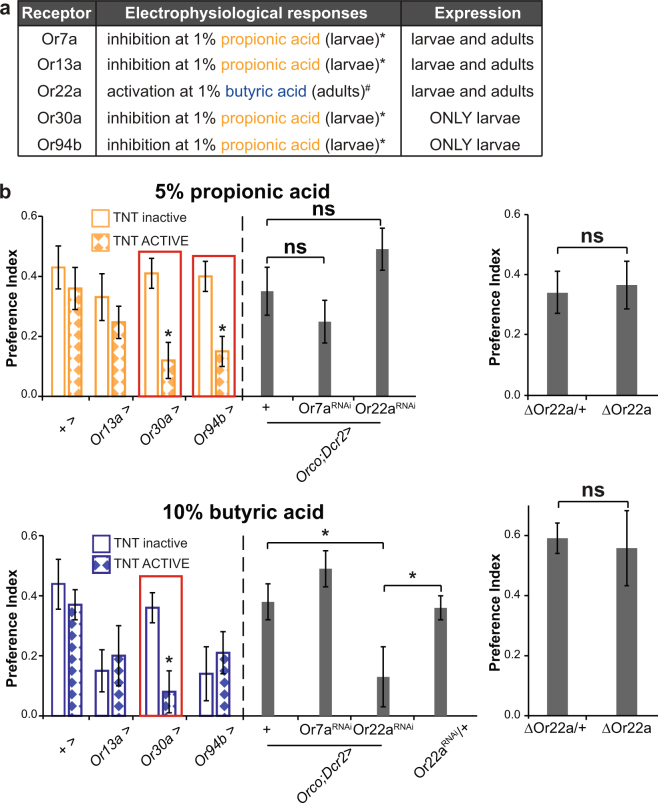
Specific olfactory receptors underlying bacterial SCFA attractive responses. (**a**) List of candidates for propionic and butyric acid detection according to electrophysiological and expression data. The “*” stands for electrophysiological data published in^22^ and “#” data published in^24^. Expression data was taken from^8,33^. (**b**) Preference index at 5% propionic (upper panel) and 10% butyric acid (lower panel). Propionic acid attraction depended on the activity of Or30a^+^ and Or94b^+^ neurons, while butyric acid behavioral responses required Or30a^+^ neurons. The neuronal activity of Or13a^+^, Or30a^+^, and Or94b^+^ neurons was abolished by means of TNT, and the expression of Or7a and Or22a was downregulated with specific RNAi lines. The “*” indicates statistically significant differences with respect of the two genetic controls (One-way ANOVA with a Duncan post-hoc test, p < 0.05). From left to right the complete genotypes are: +  > TNT inactive, +  > TNT ACTIVE, *Or13a* > TNT inactive, *Or13a* > TNT ACTIVE, *Or30a* > TNT inactive, *Or30a* > TNT ACTIVE, *Or94b* > TNT inactive, *Or94b* > TNT ACTIVE, *Orco;Dcr2* >  + , *Orco;Dcr2* > Or7a^RNAi^, *Orco;Dcr2* > Or22a^RNAi^and for the case of butyric acid +  > Or22a^RNAi^. Analysis of the deletion mutant ΔOr22a and its heterozygous control showed no statistically significant differences between the two genotypes at 5% propionic nor at 10% butyric acid (t-TEST). Data represents the average ± standard error of the mean and an average of 20 independent replicates were considered for each experimental group, except for the case of ΔOr22a that 10 independent replicates were analyzed.

A bibliographic search focused on the analysis of ORs electrophysiology in response to a panel of odorants performed by Carlson’s group^22,24^, led us to the following list of candidates for propionic and butyric acid detection in larvae: Or7a, Or13a, Or22a, Or30a, and Or94b (Fig. 2a). Neuronal inactivation of Or30a^+^ neurons significantly reduced attractive responses towards both propionic and butyric acid. On the other hand, expression of TNT on Or94b^+^ neurons only affected propionic acid responses while inactivation of Or13a^+^ neurons did not affect neither propionic nor butyric acid behavioral responses (Fig. 2b). Available GAL4 lines for Or7a and Or22a fail to drive expression at the larval stage^8,33^, although *in situ* hybridization and immunostainings with specific antibodies confirmed the expression of these two receptors in larvae^4,8^. Thus, to analyze the role of Or7a and Or22a we downregulated each OR’s expression by means of expression of RNAi lines using an *Orco*-GAL4 line. Downregulation of Or22a reduced the attractive responses towards butyric acid while leaving the response to propionic acid unaltered. However, the analysis of the Or22 deletion mutant (Δhalo) failed to confirm the role of Or22a on butyric acid perception, leaving the possibility that the reduction on the preference index observed with the RNAi might possibly be due to an unspecific effect of the tool. Lastly, Or7a downregulation did not have any significant effect on the olfactory behavioral responses to both bacterial SCFA (Fig. 2b). Published electrophysiological data confirms that Or30a^+^ and Or94b^+^ neurons can directly detect propionic acid leading to a reduction of action potential spikes^22,24^. Noteworthy, given the limitations of screening only candidate ORs and not all larval receptors, we cannot rule out further chemoreceptors being involved.

Summing up, our results point to an essential role of Or30a^+^ neurons for the olfactory perception of both propionic and butyric acid. In addition, Or94b^+^ neurons are involved in propionic acid perception specifically. Of note, Or30a and Or94b are olfactory receptors expressed exclusively at the larval stage^33^, implying that the mechanisms of olfactory detection of bacterial SCFA differ between larvae and adults.

### Propionic acid does not promote sucrose nor amino acid perception

Attractive responses towards an odorant can indicate that the compound *per se* has a beneficial effect on animal physiology. Alternatively, fast odorant responses towards volatile compounds can act as an indicator of the presence of non-volatile chemicals such as essential nutrients. Bacterial SCFA are produced from sugar fermentation and, as a consequence, SCFA detection could also indicate the presence of a rich source of sugars. In addition, in the taste system carboxylic acids are known to act on sugar sensing neurons altering sucrose perception^34^. In this context, we decided to test if the smell of propionic acid could improve sucrose perception. We focused on propionic acid because it triggers attractive responses already at 1%, a concentration that is more consistent with the amounts normally found in rotten fruit^35^.

**Figure 3:**
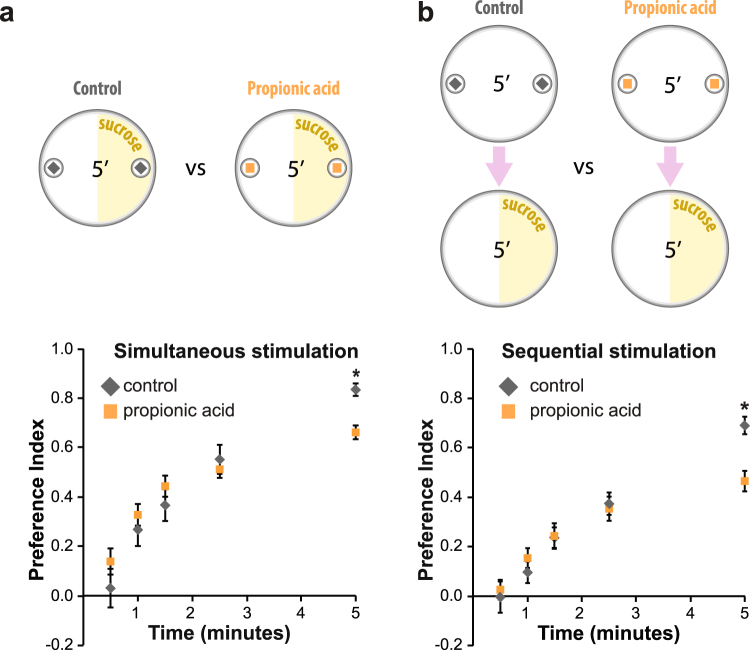
Propionic acid does not promote detection of sucrose. (**a**) Preference for 100 mM sucrose at different times in *w* ^1118^ larvae simultaneously exposed to paraffin oil (control) or 1% propionic acid. (**b**) Preference for 100 mM sucrose at different times for *w* ^1118^ larvae pre-exposed to paraffin oil (control) or 10% propionic acid. Simultaneous (**a**) or sequential (**b**) exposure to propionic acid vapors and sucrose did not promote sucrose preference but rather slightly reduced it. The “*” indicates statistically significant differences with respect of the control group (Repeated measures ANOVA with a Tukey post-hoc test, p < 0.05). Data represents the average ± standard error of the mean and 13–19 independent replicates were considered for each experimental group.

We analyzed the preference for sucrose in groups of larvae that were or were not simultaneously exposed to propionic acid vapors. In both conditions foraging larvae chose the sucrose side, and the presence of propionic acid did not increase the preference index but rather slightly reduced it (Fig. 3a). The same negative effect over sucrose perception was observed when a higher concentration of propionic acid was applied prior to the sucrose detection assay (sequential stimulation, Fig. 3b).

Flies depend on microorganisms present in the fruit like yeast and bacteria to obtain most of the essential amino acids^10,36^. As for the case of sucrose, we wondered if propionic acid could act as an indicator of an amino acid rich medium and, in doing so, might promote amino acid perception. Larvae displayed a significant attraction towards an amino acid mix, and pre-exposure to propionic acid did not alter the preference for amino acids (Supplementary Fig. S2).

In conclusion, in the conditions we tested propionic acid did not act as a stimulator for the detection of sucrose or amino acids. Although we cannot completely rule out a modulatory role of propionic acid over nutrient perception, the lack of a positive effect on sugar and amino acid detection encouraged us to directly investigate a beneficial role of propionic acid *per se* over larva physiology.

### Propionic acid improves larval survival and supports growth in a nutrient restricted diet

**Figure 4:**
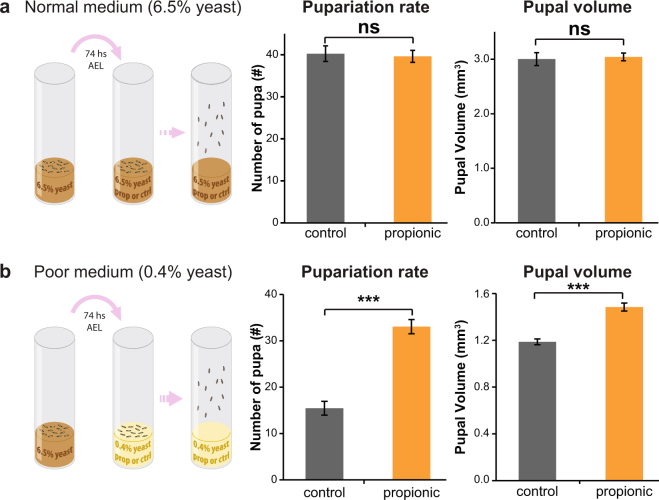
Propionic acid in the culture medium improves larval survival and supports growth. (**a**) Analysis of the pupariation rate (graph in the left) and pupal volume (graph in the right) in a normal medium (6.5% yeast). In this condition, the addition of 1% propionic acid did not affect the survival rate or the pupal size (t-TEST, n = 8 independent tubes with an average of 50 initial larvae). (**b**) Idem A but larvae were maintained in a normal medium until early L3 stage and then transferred to a poor medium (0.4% yeast). In a poor condition, the presence of 1% propionic acid significantly increased the pupariation rate and the pupal volume (***p < 0.0001 t-TEST, n = 22 independent tubes with an average of 50 initial larvae).

As a first approach to reveal any potential beneficial effect of propionic acid over larval physiology, we performed developmental studies in different rearing conditions. To this end, we exposed larvae to different nutritional conditions from the beginning of L3 stage (74 h AEL), a critical period when larvae show the greatest growth rate in order to rapidly achieve the minimal weight that will ensure progression of development^37^. Noteworthy, early L3 larvae are also attracted toward propionic acid (Supplementary Fig. S3a). In a standard medium rich in carbohydrates and yeast, the addition of propionic acid did not affect pupariation rate, nor the final size achieved to the pupal stage (Fig. 4a). However, a standard medium provides an excess of all the nutrients larvae need to develop normally, and in those conditions a beneficial role of propionic acid could be easily masked. Therefore, we resolved to investigate the effect of propionic acid in a non-nutritious and in a nutrient-restricted medium. In a non-nutritious medium (1% agar) the survival of early L3 was strongly impaired, and 24 h post-transference to this medium the average mortality was of 75% and 58% in the control and propionic acid-supplemented condition, respectively. The addition of propionic acid in the culture medium significantly increased larval survival although the effect was modest and after 8 days all larvae were dead (Supplementary Fig. S3b). In a poor medium (absence of carbohydrates and a minimum amount of yeast) and without propionic acid, less than 30% of the larvae were able to grow and reach pupariation (an average per tube of 14 survivors out of 50 larvae). Strikingly, supplementing the medium with propionic acid strongly improved larval development, leading to an increase of larval survival and pupariation rate (Fig. 4b, left graph). Moreover, of the larvae that reached pupariation those reared with propionic acid were notably bigger, suggesting that in these conditions propionic acid promotes larval growth (Fig. 4b, right graph).

### Propionic acid is an appetitive odor that promotes feeding behaviors in a poor medium

**Figure 5:**
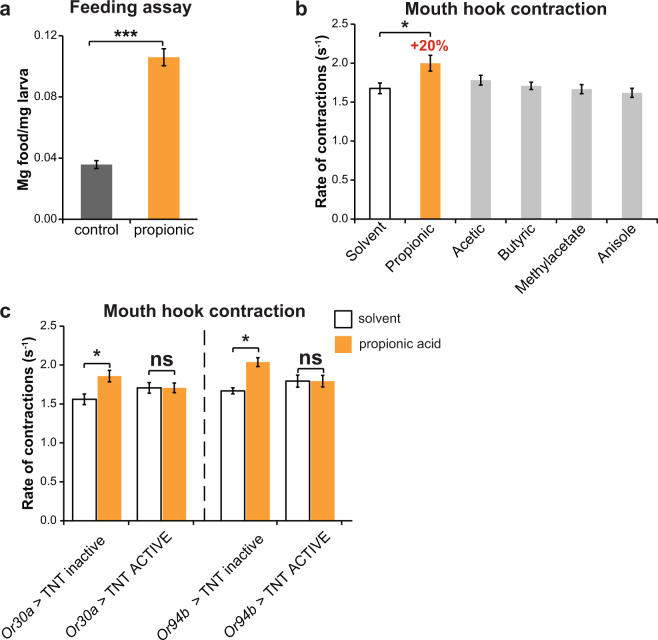
The smell of propionic acid is sufficient to promote feeding behaviors in a low nutrient medium. (**a**) Feeding assay of *w* ^1118^ larvae in the context of a poor medium (0.4% yeast). Foraging larvae ingested significantly more food in the presence of 1% propionic acid (***p < 0.0001 t-TEST, n = 15 independent replicates). (**b**) Rate of mouth hook contractions in the presence of different odorants for*w* ^1118^ larvae. Only propionic acid elicited a statistically significant increase of mouth hooks contractions of 20% with respect to the control (One-way ANOVA with a Tukey post-hoc test p < 0.05, n = 15). (**c**) The propionic acid-stimulated increase on mouth hook contractions required functional Or30a^+^ and Or94b^+^ neurons (Two-way ANOVA followed by a Tukey post-hoc test, p < 0.05). Data represents the average ± standard error of the mean and a minimum of 15 independent replicates were considered for each experimental group.

The observation that larvae were able to grow bigger in a propionic acid-supplemented medium prompted us to investigate if propionic acid could promote feeding behaviors. To test this hypothesis, we exposed early L3 larvae to a poor medium containing a blue dye with or without propionic acid for 30 minutes, after which we analyzed the quantity of food ingested by absorbance at 625 nm. Larvae exposed to the propionic acid supplemented medium ingested almost 3 times more food than the control groups (Fig. 5a). Interestingly, this positive effect is specific of the larval stage since propionic acid in adult flies failed to promote feeding, consistent with the innate aversion towards the smell of propionic acid at the adult stage (Supplementary Fig. S4).

In order to test if the effect over feeding was driven specifically by odor perception, we studied larval feeding behavior in the presence of propionic acid vapors. Brief exposure to appetitive olfactory cues can trigger an increase of mouth hook contractions even in well fed larvae^38^. We exposed well fed early L3 larvae to odorants vapors in the context of a poor medium, after which we recorded and counted mouth hook contractions. In the presence of the solvent, the mouth hook contraction rate was of an average of 1.68 contractions per second. Strikingly, exposure to propionic acid vapor increased the contraction of mouth hooks by 20% in control larvae while no promotion effect was evident in *Orco* null mutants (Fig. 5b and Supplementary Fig. S5a). This effect depended on an accurate olfactory perception of propionic acid, since no increase of mouth hook contractions was observed in larvae expressing TNT in Or30a^+^ or Or94b^+^ neurons (Fig. 5c). Interestingly, the effect over feeding behavior was specific of propionic acid since other SCFA (acetic and butyric acid), an ester with the same molar mass (methylacetate), or an unrelated compound but with the same vapor pressure (anisole), failed to promote an increase on mouth hook contractions (Fig. 5b). All of these compounds are detected by foraging larvae and they triggered attraction on a chemotaxis assay to a similar or even higher extent than propionic acid (Table 1). It is worth noting that the effect of propionic acid on mouth hook contraction was not observable when the exposure was performed in the context of either a normal or a non-nutritious medium (Supplementary Fig. S5b,c), mimicking the effect observed on larval survival in different rearing conditions.

**Table 1:** Odorants used in Fig. 5b. Basic chemical properties and the attractive responses.

Odorant	Chemical group	Molar mass (g/mol)	Vapor pressure (kPa)*		Chemotaxis responses^#^		
					Preference Index (PI)	t TEST PI = 0	n
Acetic acid	SCFA	60	2.09	Same chemical group than PA	0.83 ± 0.03	**p < 0.0001**	20
Propionic acid (PA)	SCFA	74.08	0.47		0.52 ± 0.06	**p < 0.0001**	22
Butyric acid	SCFA	88.1	0.22	Same chemical group than PA	0.12 ± 0.08 (at 1%)	p = 0.1440	20
					0.67 ± 0.04 (at 10%)	**p < 0.0001**	20
Methyl acetate	Ester	74.08	23.1	Same molar mass than PA	0.89 ± 0.03	**p < 0.0001**	20
Anisole	Aromatic	108.01	0.47	Same volatility than PA	0.51 ± 0.06	**p < 0.0001**	20

*Vapor pressure of pure odorants at 25 °C except for methyl acetate that is at 20 °C.

^#^Preference Index correspond to chemotaxis assay of control *w* ^1118^ larvae using 1% of each odorant with the exception of butyric acid that was tested at 1 and 10%. “t-TEST PI = O” indicates p value for a t-TEST with the null hypothesis PI = 0. N indicates the number of independent replicates for each condition.

### Propionic acid does not induce feeding behaviors in *D. suzukii*, a related species that exploits a different ecological niche than *D. melanogaster*

During the transition from green to ripe and overripe fruit, diverse fermentation processes increase the levels of certain organic acids^39^. In particular, bacterial SCFA are mostly produced at the last stages of fermentation probably because the increasing pH leads to the growth of bacteria able to produce propionic and butyric acid^19,20^.

**Figure 6:**
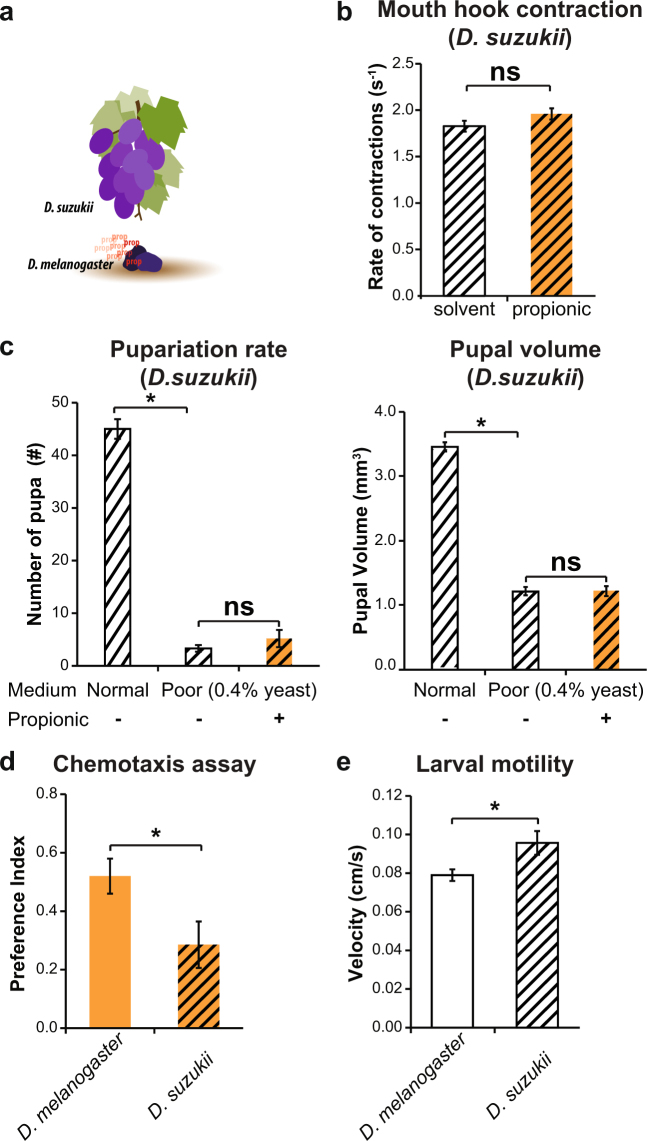
Propionic acid does not promote feeding behaviors in *Drosophila suzukii*. (**a**) Schematic drawing illustrating the differences on ecological niche occupied by *Drosophila melanogaster* and *D. suzukii*. *D. melanogaster* adults have a preference for overripe, rotten, and fermenting fruit, where the levels of propionic acid are higher. In contrast, *D. suzukii* adults attack fresh fruit which present lower levels of fermentation products as propionic acid. (**b**) Rate of mouth hook contractions of early *D. suzukii* foraging larvae in the context of a poor medium (0.4% yeast). Contrary to what was observed in *D. melanogaster*, propionic acid did not trigger an increase of mouth hooks contractions in *D. suzukii* larvae (t-TEST, n = 15 independent replicates). (**c**) Pupariation rate (graph in the left) and pupal volume (graph in the right) for *D. suzukii* larvae in a normal medium (6.5% yeast) or a poor medium (0.4% yeast) supplemented or not with 1% propionic acid. *D. suzukii* larvae survived and grew significantly less in a poor medium compared to a normal medium, and this effect was not reversed by the addition of propionic acid (One-way ANOVA followed by a Tukey post-hoc test, p < 0.05, n = 6–12 independent tubes with an average of 50 initial larvae). (**d**) Preference index for 1% propionic acid in a chemotaxis assay. *D. suzukii* larvae showed a reduce attraction to the smell of propionic acid compare to *w* ^1118^ *D. melanogaster* larvae (*p = 0.0189 t-TEST, n = 21). (**e**) Mean larval velocity of *w* ^1118^ (*D. melanogaster*) and wild type *D. suzukii* in the absence of odorants. *D. suzukii* foraging larvae were statistically significant faster than *D. melanogaster* (*p = 0.0258 t-TEST, n = 20). In all graphs orange and empty bars indicate presence and absence of 1% propionic acid, respectively. Striped bars represent data from *D. suzukii*.

*Drosophila melanogaster* feed, lay eggs, and develop in overripe, rotten, or fermenting fruit^40^ where the levels of bacterial SCFA are higher. On the contrary, the spotted-wing fly *Drosophila suzukii* is capable of colonizing ripening fruit (Fig. 6a) and, in doing so, causes serious crops damage^28^. In this context, we wondered if *Drosophila suzukii* larvae, which are exposed to ripening fruits with lower levels of bacterial SCFA, would behave differently towards propionic acid odor. We hypothesized that, given the lower exposure to propionic acid in its natural environment, *D. suzukii* larvae will not display propionic acid-induced feeding behaviors. This is indeed the case, as we found that the rate of mouth hook contractions in *D. suzukii* larvae remained unaltered after propionic acid exposure in the context of the same low-nutrient medium used for *D.melanogaster* experiments (Fig. 6b). Exposure of *D. suzukii* larvae to this medium significantly reduced larval survival and growth and this was not reversed by a propionic acid supplement (Fig. 6c). These results confirm that the low-nutrient medium also represents a poor nutritional condition for *D. suzukii* and suggest that in this species propionic acid does not have a clear beneficial effect over larval development. *D. suzukii* were more sensitive to this low-nutrient medium with only yeast as an energy source (28% larval survival in *D.melanogaster* vs 6,67% in *D.suzukii*), highlighting again the different nutritional requirements for both species. Moreover, the attraction towards propionic acid in a chemotaxis assay was significantly lower in *D. suzukii* compared to *D. melanogaster* larvae (Fig. 6d), and this was evident even considering that *D. suzukii* had a higher basal larval motility than *D. melanogaster* larvae (Fig. 6e).

To conclude, for the related species *D. suzukii* that exploits a different ecological niche with lower bacterial SCFA concentration, propionic acid results less attractive, does not promote feeding behaviors, and does not improve larval survival and growth.

## Discussion

In this work, we characterized attractive responses towards propionic and butyric acid in *D. melanogaster* larvae. We have identified the chemoreceptors essential for those olfactory attractive responses and we described the beneficial effects of propionic acid on larval physiology. In particular, we demonstrated that odor perception of propionic acid can act as an orexigenic signal triggering feeding behavior in a nutritionally-deficient context.

Propionic and butyric acid are SCFA produced after carbohydrate fermentation performed by bacteria of the genus *Propionibacterium*, *Veillonella*, *Bacteroides*, *Clostridium*, *Butyrivibrio*, *Bacillus*, *and Lactobacillus* ^29,41^. *Propionibacterium cyclohexanicum* and *Lactobacillus diolivorans* are naturally present in several fruits^42,43^. Plus, several species of *Bacteroides* and *Bacillus* have been identified as part of the adult fly microbiota^44^. Interestingly, *Drosophila* maintains its internal microbiota by frequent ingestion of bacteria present in their medium^45^, suggesting that *Bacteroides* and *Bacillus* in the gut are indeed obtained from the fermenting fruit.

In the last years, several studies demonstrated that bacteria-derived odors modify fly behavior. Pathogenic bacteria present in carnivore feces produce phenol and this odor induces egg-laying aversion^46^. Bacterial odors mediate social attraction in adults and larvae^47^. Several amines, products of bacterial protein degradation, are attractive to flies and trigger oviposition^48^. A recent study showed that an interactive multispecies-microbiota leads to acetic acid production and this SCFA together with its metabolic derivates are responsible for the higher attraction towards the complex mix of microorganisms^49^. It would be interesting to test if other mix of microorganisms could give raise to different metabolites, like propionic acid. Being able to rapidly detect a heterogeneous microbial community could be beneficial to replenish gut microbiota^45^. Furthermore, propionic acid production could have an additional protective role since it reduces fungal growth^50^. Future studies should consider the influence of microbial composition in the diet and the consequent levels of propionic acid produced.

In the case of propionic acid we found that olfactory attractive responses correlate with an increase in feeding behavior specifically at the larval stage. This orexigenic effect was only evident when larvae were exposed to a poor nutritional medium, suggesting that in a condition of nutrient excess other food odors may be sufficient to trigger feeding. As we detailed before, propionic acid is a byproduct of bacterial sugar fermentation. Fast detection of the volatile compound propionic acid could guide larvae toward a sugar-rich spot in a nutrient-poor context. However, excessive levels of propionic acid could be an indicator that bacteria are depleting the scarce sugar sources. On the other hand, bacteria itself could be a natural source of food^51^. Additionally, in mammals SCFA have been extensively characterized as important internal metabolites. Internal SCFA can modulate the insulin signaling pathway, affecting metabolic pathways such as lypolisis and lipid storage. In the gut, propionic acid can be converted into glucose^14^. In this regard, it is possible that in a context of reduce nutrients propionic acid incorporated from the diet may act itself as an energy source in *Drosophila melanogaster* larvae. In the fruit fly, the gut commensal bacterium *Acetobacter pomorum* produces acetic acid and this affects development and metabolism^52^. Our results prove that propionic acid can also modulate larval development, since propionic acid in the culture medium increases larval survival and promotes growth. In addition to the putative effect of propionic acid as an internal signal modulating larval growth, in this paper we demonstrate that propionic acid vapors act as an exogenous orexygenic signal. Thus, odor-driven behavior could constitute the first step on the overall beneficial effect of propionic acid on larval development. Furthermore, the promotion of feeding behavior is specific of propionic acid and it was not observed when larvae were exposed to acetic acid vapors. In this sense, our results describe a new beneficial effect of a bacterial SCFA that could save larvae from death when nutrients are scarce. These results prompted us to study if this role of propionic acid was conserved in other *Drosophila* species such as *Drosophila suzukii*.

*Drosophila suzukii* females possess a morphologically unique ovipositor that allows perforation and penetration of fruit skin^28^. Thanks to this evolutionary innovation, *D. suzukii* is a unique species in the melanogaster group because it lays eggs in the early stages of fruit maturation^40^ and in doing so, it damages fresh ripe fruit causing important agricultural damage^53^. Since propionic acid is mostly produced at the last stages of fruit fermentation^19,20^, *D. suzukii* attacking fresh fruit will be less exposed to this bacterial SCFA compared to its related species *D. melanogaster*. Consistent with this prediction, we found that propionic acid is less attractive for *D. suzukii* larvae and it does not promote feeding behaviors in this species.

In *D. melanogaster* larvae we demonstrated that both propionic acid-driven behavior, *i.e*. attraction and increase of feeding behavior, depends on the correct activity of Or30a and Or94b^+^ neurons. Contrary to the case of other receptors detecting volatile compounds produced during fermentation, *D. suzukii Or30a* and *Or94b* do not display clear signs of selective pressures^54,55^ and the alignment of protein sequences show a high level of conservation between *D.suzukii* and *D. melanogaster* receptors (93% and 90% protein identity for Or30a and Or94b, respectively). However, although the molecular evolution analysis suggests that *D. suzukii Or30a* and *Or94b* genes will give rise to similar proteins than those of *D. melanogaster* we don’t know if they will be correctly expressed in the specific OSNs at the larval stage. Future studies should investigate if *D. suzukii* Or30a^+^ and Or94b^+^ neurons can detect or not propionic acid in larvae. The available data support the notion that *D. suzukii* larvae most probably are able to detect propionic acid but they respond differently than *D. melanogaster*. We propose that the shift to a new ecological niche in *D. suzukii* signified a change on the hedonic value conferred to propionic acid and, as a consequence, a reduction or loss of the attractive behavioral responses towards this bacterial SCFA.

Innate olfactory behavior towards SCFA in *D. melanogaster* larvae are opposite to adult behavioral responses. We here confirmed the innate aversion towards moderate concentrations of propionic acid and we demonstrated that this SCFA does not promote feeding in adults, emphasizing the different roles of propionic acid along *D.melanogaster*’s life cycle. The aversion towards the smell of SCFA in adults seems to be an escape response to acid solutions that can be highly toxic^21,56^. We found that larvae are attracted to the smell of propionic and butyric acid even when they were exposed to pure odorants during more than one hour with no signs of damage, indicating that larvae are more tolerant to highly acid solutions. Prolonged exposure to low pH media in overripe fruit throughout all larval development might require an increased tolerance to acidity.

SCFA olfactory responses in adult flies depend on the direct detection of the hydronium by the acid receptor Ir64a, the detection of the carboxylic chain by Ir75a, but also some ORs and Ir76b^6,7,21,24^. Both Ir64a and Ir75a require, to function, the coreceptor Ir8a^6,57^, a receptor that is not expressed at the larval stage^6^. At the same time, we demonstrated that attractive responses to SCFA in larvae requires olfactory receptors that are exclusively expressed at the larval stage, *i.e*. Or30a and Or94b, although given our restricted screen we cannot rule out the relevance of further chemoreceptors. As a result, SCFA-evoked aversive and attractive behavioral responses rely on chemoreceptors expressed exclusively at the adult and the larval stages, respectively. In this regard, stage-specific SCFA detection systems could be partly responsible for the opposite behaviors at each stage of the fly’s life cycle. To complete the panorama of SCFA perception, future studies should take into account the role of taste perception. Our preliminary analysis points to the coreceptor Ir25a as a putative relevant taste receptor for both propionic and butyric acid perception. In larvae, Ir25a is expressed in several taste cells^58^ and in DO cells dedicated to cool sensation^59^. In our chemotaxis assay, null mutants for Ir25a or inactivation of Ir25a^+^ cells displayed a lower attraction towards propionic and butyric acid (Supplementary Fig. S6). Future studies should consider an optimized behavioral paradigm to study taste perception at the larval stage. Additionally, a broader analysis of chemoreceptors to study the contribution of other families of taste receptors like GRs would significantly improve our understanding of SCFA perception in flies.

In conclusion, our results in context with published data manifest that propionic acid is a complex signal that triggers opposite behaviors in larvae and adults, and that the larval responses are not conserved in the related species *D. suzukii*. Even more, we here demonstrated that olfactory detection of propionic acid promotes feeding when nutrients are scarce, highlighting the important role of propionic acid in a challenging situation for larval survival.

## Materials and Methods

### Fly rearing and stocks

Flies were grown and maintained at 25 °C in vials containing cornmeal (6.5% m/v), agar (1% m/v), Tegosept (Apex) antifungic (3% v/v), and yeast (6.5% m/v) under 12:12 h light:dark cycles. Noteworthy, our standard food medium does not contain propionic acid. For chemotaxis assays, larval motility, and sucrose and amino acid detection experiments, 4 days old foraging larvae (between 90-100 h AEL, AEL = after egg laying) were used, unless indicated otherwise. For larval development, feeding, chemotaxis assay on Supplementary Fig. S3a, and mouth hook contraction’s experiments, eggs were collected onto an agar plate with alive-yeast paste for 4 hours to obtain synchronized larvae. 24 hours later, first instars larvae were transferred to standard food vials until early third-instar larvae (74 ± 2 h AEL). All stocks used in this study were described previously: *w* ^1118^, *Orco* ^2^, *Ir8a* ^1^, *Ir*2*5a* ^2^, *Orco*-GAL4, *Ir25a*-GAL4, UAS-TNT inactive (an inactive mutant of the tetanus neurotoxin light chain that does not block chemical synapses), and UAS-TNT ACTIVE were gently provided by Richard Benton. Δhalo flies were kindly supplied by Silche Sachse. *Ir76b* ^2^ (#51310), *Ir76b*-GAL4 (#51311), *Or13a*-GAL4 (#9945), *Or30a*-GAL4 (#9960), and *Or94b*-GAL4 (#23917), were obtained from the Bloomington Stock Center. UAS-Dicer2 (#60009), UAS-Or7a^RNAi^ (#107874), and UAS-Or22a^RNAi^ (#49835) were obtained from the Vienna RNAi Stock Center. Wild type *Drosophila suzukii* were a kind gift from Patricia Gibert.

All strains have been backcrossed to an isogenic *w* ^1118^ strain for five generations with the exception of Δhalo flies and the wild type strain of *Drosophila suzukii*.

In order to exclude potential effects of different nutrients during early development and focus on acute effects of propionic acid at the 3^rd^ instar stage, the same medium was used to raise *D. melanogaster* and *D. suzukii* flies.

### Odor stimulation

All odorants tested were purchased from commercial sources (Sigma, http://www.sigma-aldrich.com and Thermo Fisher Scientific https://www.fishersci.com/us/en/brands/I9C8LQ1I/acros-organics.html). Odorants were diluted in paraffin oil (Sigma, 8012-95-1). For behavioral analysis, 20 μl of the appropriate dilution or 3 μl of pure odorants were deposited in a filter paper placed inside a plastic cap.

### Chemotaxis assay

Chemotaxis two choice assays were performed as described before^23^. Briefly, 50 foraging larvae were placed in the center of a 94 mm agarose petri dish where they were given the choice between an odorant dilution and paraffin oil. Larvae were recorded for 5 minutes (or 10 minutes in the case of 74 h AEL larvae) after what the numbers of larvae in different zones were counted. An “odorant zone O” was established within a 58 mm circumference surrounding the odor source while the respective circumference around the solvent corresponded to the “solvent zone S”. The preference index was calculated as follows: (#larvae in O - #larvae in S)/ (#larvae in O + #larvae in S). Since early L3 larvae (74 ± 2 h AEL) are significantly smaller than 4 days old larvae (between 90-100 h AEL), they need more time to explore the plate, so the preference index was calculated after 10 minutes.

Innate olfactory responses in male and female adults were studied using a modified Y-maze device as previously described^60^.

### Sucrose and amino acid detection experiments

Sucrose (Euromedex 200-301) or the MEM amino acid mix for cell culture (ThermoFisher 11130) were solubilized in 1% agarose and poured into one side of a 94 mm petri dish. Once solidified, the other side of the dish was filled with plain agarose.

The effect of propionic acid over sucrose detection was studied by simultaneous or sequential stimulation with both chemicals in *w* ^1118^ larvae. For the case of simultaneous stimulation (Fig. 3a), the “control group” had filter papers containing paraffin oil in both corners of the 100 mM sucrose/plain agarose plate, while the “propionic group” had 1% propionic acid at both sides. For sequential stimulation (Fig. 3b), a higher concentration of propionic acid was used foreseeing that a stronger pre-exposure could be required to reveal a positive interaction over sucrose detection. Larvae were pre-exposed for 5 minutes to 10% propionic on a plain agarose plate, then collected, rinsed, and transferred to a 100 mM sucrose/plain agarose plate.

The effect of propionic acid over amino acid detection was studied in *w* ^1118^ larvae only in the sequential stimulation condition and following the same procedure than for sucrose detection experiments but using 2X MEM amino acid mix.

In all the cases, the number of larvae found on the plain agarose (C) and sucrose or amino acid areas (S/Aa) was counted after 0.5, 1, 1.5, 2.5, and 5 minutes. The larvae found at a distance of 0.5 cm on either side of the dividing line were not included in the index calculation. A preference index was calculated as follows: (#larvae in S/Aa - #larvae in C)/ (#larvae in S/Aa + #larvae in C).

### Larval survival, pupariation rate, and pupal size

*w* ^1118^ or wild-type *D. suzukii* larvae developed in a standard medium until early L3 stage (74 ± 2 h AEL) and, at that point, they were transferred to different media. In “normal medium” experiments (Figs 4a and 6c left bar), 50 larvae were transferred to new tubes containing the same normal medium (6.5% yeast), while in “poor medium” experiments (Figs 4b and 6c right bars), they were transferred to an agar tube containing only 0.4% alive-yeast. A control and a 1% propionic acid supplemented conditions were analyzed. The number of pupa was counted and the pupal size was calculated as previously described^61^.

In order to test the effect of propionic acid in a non-nutritious medium, 50 early L3 stage *w* ^1118^ larvae were transferred to agar plates supplemented or not with 1% propionic acid and the number of dead larvae were counted twice per day. Each plate was considered as an independent replicate.

### Feeding assay and mouth hook contraction’s analysis

Early L3 stage *w* ^1118^ larvae were transferred to 0.4% alive-yeast plates containing 0.5% Brilliant blue FCF dye (Sigma, 3844-45-9) supplemented or not with 1% propionic acid. After 30 minutes, 2 groups of 12 larvae per plate were recovered, rinsed in PBS, dried in a Whatman paper and frozen immediately at −80 °C. The amount of food ingested was determined by absorbance measurement of FCF blue dye at 625 nm. To avoid larval weight effects, values were expressed as mg of ingested food/mg larvae. For adult feeding analysis, young *w* ^1118^ (2-5 days old) virgin males and females were transferred and maintained for 8 hours in 0.4% alive-yeast tubes containing 1% of the same blue dye, after what they were washed, dried, frozen, and quantified as for the case of larvae. Each tube containing 15 male or female flies was considered as an independent replicate.

To study mouth hooks contractions we adapted a protocol previously described^38,62^. Briefly, 15 early L3 stage larvae were pre-fed with a poor alive-yeast (0.4%) liquid solution for a total of 30 min of which the last 10 minutes were in the presence of 20 μl of 1% odorant (except butyric acid that was tested at 10%) or paraffin oil in the treated and control group, respectively. After stimulation, larvae were rinsed and transferred to a 35 mm petri dish containing a poor alive-yeast-agar paste, also named “liquid food”. After 1 min acclimation, larvae were videotaped for 4 min. Mouth hook contractions were counted manually over a period of 30 seconds, and the average of 2–4 larvae per video was considered as a single replicate.

For the analysis of mouth hook contractions in a normal medium, larvae were pre-fed with a 10% alive-yeast liquid solution and then transferred to a rich glucose-agar paste. In the case of non-nutritious medium, instead of the 30 min pre-feeding time, larvae were left in 100 μl of distillated water, and the recording of mouth hook contractions was performed in a plain agar paste.

### Larva motility

Locomotor activity of foraging larvae in the absence of odorants was tracked and recorded for 5 min at a sampling rate of 0.5 frames/s using Ethovision Pro (Noldus) tracking software. For each plate the average of 3 larvae was considered as a single replicate, and a total of 20 independents plates were analyzed per group.

### Statistical analysis

Statistical analyses were performed with the InfoStat package version 2009 (Grupo InfoStat, FCA, Universidad Nacional de Cordoba, Argentina), with the exception of the Gehan-Breslow-Wicoxon test that was conducted using GraphPad Prism 7.3 software (Graphpad). Normality was tested using Shapiro-Wilks test and the homogeneity of variance was assessed with Levene’s test. p < 0.05 was considered statistically significant. In the case of Repeated measures ANOVA (Fig. 3), a Box’s test for homogeneity of variance and covariance matrices, and Mauchly’s sphericity test were performed. For the analysis of mortality curves in Supplementary Fig. S3b, each independent replicate, *i.e*. each plate with 50 initial larvae, was adjusted to a Gompertz non-linear regression model, and the median lethality per plate was estimated. The lethality curves for each condition were compared with a Gehan-Breslow-Wilcoxon survival test using the estimated median values. This statistical analysis compares the mortality curves as a whole instead of a comparison point by point. The statistical tests and special requirements for each experiment are detailed in the legends of the corresponding figures.

## Electronic supplementary material

Supplementary figures and legends
