# Supplementary material for: Olfactory detection of a bacterial short-chain fatty acid acts as an orexigenic signal in Drosophila melanogaster larvae
Source: Sci Rep. 2017 Oct 27;7:14230. doi: 10.1038/s41598-017-14589-1 (PMC5660182; doi:10.1038/s41598-017-14589-1)
Supplement: Supplementary file 1 — Supplementary figures and legends [file 41598_2017_14589_MOESM1_ESM.doc]

**Supplementary Information**

**Olfactory detection of a bacterial short-chain fatty acid acts as an orexigenic signal in *Drosophila melanogaster* larvae**

Ana Depetris-Chauvin^1,+^, Diego Galagovsky^1^, Charlene Chevalier^1^, Gerard Maniere & Yael Grosjean^1,*^

^1^ Centre des Sciences du Goût et de l'Alimentation, AgroSup Dijon, CNRS, INRA, Univ. Bourgogne Franche-Comté, F-21000 Dijon, France.

^+^ Present address: Department of Evolutionary Neuroethology, Max Planck Institute for Chemical Ecology, 07745 Jena, Germany.

^*^ Correspondence and requests for materials should be addressed to Y.G. (yael.grosjean@u-bourgogne.fr)

**Supplementary Figure 1.** Preference index for the control line *w^1118^*, the null mutant *Orco^-/-^*, TNT control lines, and inactivation of Orco^+^ neurons at 1% propionic acid. Mutation of *Orco* significantly reduced the preference index for propionic acid (t-Test p= 0.0036, n= 20-22), but a residual attraction is still evident (t-Test with null Hypothesis PI = 0, p = 0.0003). At 1% the preference index of control line *+* > TNT ACTIVE was extremely low to perform future analysis. (Two-way ANOVA, p< 0.05. ns = non-statistically significant differences between the control lines *+* > and the *Orco* > lines for both TNT inactive or TNT ACTIVE crosses). From left to right the complete genotypes are: *w^1118^*, *Orco^-/-^* = *Orco^2^* mutant, *+* > TNT inactive, *+* > TNT ACTIVE, *Orco* > TNT inactive, and *Orco* > TNT ACTIVE.

**Supplementary Figure 2.** Preference for an amino acids solution (2X MEM medium) at 5 minutes for *w^1118^* larvae pre-exposed to paraffin oil (control) or 1% propionic acid. Both groups displayed significant attraction towards the amino acids mix (t-Test with null Hypothesis PI = 0, p = 0.0021 and p = 0.0003 for control and propionic group, respectively). The presence of propionic acid did not alter the detection of amino acids (t-TEST, n = 20).

**Supplementary Figure 3.** (**a**) Early L3 stage *w^1118^* larvae (74 h AEL) are attracted towards 1% propionic acid (t-Test with null Hypothesis PI = 0, p = 0.0001, n = 16). (**b**) Mortality (relative frequency) curves of *w^1118^* larvae exposed from early L3 stage (74 h AEL) to a non-nutritious medium (1% agar) supplemented or not with 1% propionic acid. In the “propionic acid” condition a small but statistically significant improvement of larval survival was observed. (Gompertz regression followed by a Gehan-Breslow-Wilcoxon survival test. n = 8 for control and n = 9 for propionic condition).

**Supplementary Figure 4.** (**a**) Adult feeding assay in the context of a poor medium (0.4% alive-yeast). The presence of 1% propionic acid in the culture medium did not alter the amount of food ingested in *w^1118^* males nor in females (non-parametric test Kruskal-Wallis, n = 10-12 independent replicates). (**b**) Preference index (PI) at 1% propionic acid in *w^1118^* adult male and female flies. Mean aversive responses in males and females were statistically different from 0 (p = 0.0002 for males and p = 0.0009 for females, t-Test with null Hypothesis PI = 0). Aversive responses in males and females were not statistically different (non-parametric test Kruskal-Wallis, n = 30 independent replicates).

**Supplementary Figure 5.** (**a**) Propionic acid did not modify the rate of mouth hook contractions in *Orco* null mutant larvae in the context of a poor medium (t-TEST, n = 15). (**b-c**) In control larvae *w^1118^*, the presence of propionic acid in a normal medium (**b**) or in a non-nutritious medium (**c**) did not trigger an increase of mouth hook contractions (t-TEST, n = 15 for each case).

**Supplementary Figure 6.** Mutation of the coreceptor Ir25a or inactivation of Ir25a^+^ sensory neurons significantly reduced the attraction towards propionic (left) and butyric acid (right). For the null mutant *Ir25a^-/-^* the “*” indicates statistically significant differences with the control group *w^1118^* (t-TEST, p< 0.05). *Ir25a^-/-^*= *Ir25a^2^* mutant. In the case of TNT neuronal inactivation, the “*” indicates statistically significant differences with respect of the two genetic controls, UAS-TNT ACTIVE/+ and the specific driver line crossed with TNT inactive (Two-way ANOVA with a Duncan post-hoc test, p< 0.05). From left to right the complete genotypes are: *w^1118^*, *Ir25a^-/-^* = *Ir25a^2^* mutant, *+* > TNT inactive, *+* > TNT ACTIVE, *Ir25a* > TNT inactive, and *Ir25a* > TNT ACTIVE. Data represents the average ± standard error of the mean, and an average of 20 independent replicates was considered for each experimental group.
